# DEPRESSIVE SYMPTOMS AND MULTIMORBIDITY IN CONTEXT: INTERPERSONAL, SOCIOCULTURAL, AND TEMPORAL EFFECTS

**DOI:** 10.1093/geroni/igac059.714

**Published:** 2022-12-20

**Authors:** Irina Mindlis, Tracey Revenson

**Affiliations:** The Graduate Center, City University of New York, New York City, New York, United States; The Graduate Center, City University of New York, New York, New York, United States

## Abstract

Among older adults with multimorbidity (MM), disease-related stressors (e.g., pain) are associated with greater depressive symptoms. However, the contextual factors influencing this relationship remain understudied. We explored the moderating effects of interpersonal, sociocultural, and temporal factors as buffers of this relationship. Adults ≥ 62 years with MM (n=366) recruited through a national health volunteer registry and an online panel platform completed validated scales assessing diagnoses, disease-related stressors (pain intensity, subjective cognitive function, physical function, somatic symptoms), depressive symptoms. Potential moderators: age, expectations regarding aging, perceived social support, and difficulty affording medications (proxy for SES). Data were analyzed with structural equation modeling. Participants were 62-88 years old and living with many illnesses (M = 3.5; range: 2-9); 15% reported moderate-to-severe depressive symptoms. Among those with low social support, the effect of disease-related factors on depressive symptoms was greater (B = .70, SE = .06, p <.001) than for those with high social support (B = .46, SE = .06, p < .001)]. The negative effect of disease-related factors on depressive symptoms was stronger for those with worse expectations of aging (B = .68, SE = .07, p <.001), compared to those with more positive expectations (B = .47, SE = .06, p < .001). Age and difficulties affording medications were not significant moderators. Among older adults with MM, garnering social support and addressing low expectations for old age may be key to preventing the detrimental effect of MM on mental health.

